# Pathogenic variants in *HTRA2* cause an early-onset mitochondrial syndrome associated with 3-methylglutaconic aciduria

**DOI:** 10.1007/s10545-016-9977-2

**Published:** 2016-09-30

**Authors:** Monika Oláhová, Kyle Thompson, Steven A. Hardy, Inês A. Barbosa, Arnaud Besse, Maria-Eleni Anagnostou, Kathryn White, Tracey Davey, Michael A. Simpson, Michael Champion, Greg Enns, Susan Schelley, Robert N. Lightowlers, Zofia M. A. Chrzanowska-Lightowlers, Robert McFarland, Charu Deshpande, Penelope E. Bonnen, Robert W. Taylor

**Affiliations:** 1 Wellcome Trust Centre for Mitochondrial Research, Institute of Neuroscience, The Medical School, Newcastle University, Framlington Place, Newcastle upon Tyne, NE2 4HH UK; 2 Division of Genetics and Molecular Medicine, King’s College London School of Medicine, London, UK; 3 Dept of Molecular and Human Genetics, Baylor College of Medicine, Houston, TX USA; 4 Electron Microscopy Research Services, Newcastle University, Newcastle upon Tyne, UK; 5 Department of Inherited Metabolic Disease, Guy’s and St Thomas’ NHS Foundation Trusts, Evelina London Children’s Hospital, London, UK; 6 Lucile Packard Children’s Hospital Stanford and Stanford University Medical Center, Palo Alto, CA USA; 7 Clinical Genetics Unit, Guys and St Thomas’ NHS Foundation Trust, London, UK

## Abstract

Mitochondrial diseases collectively represent one of the most heterogeneous group of metabolic disorders. Symptoms can manifest at any age, presenting with isolated or multiple-organ involvement. Advances in next-generation sequencing strategies have greatly enhanced the diagnosis of patients with mitochondrial disease, particularly where a mitochondrial aetiology is strongly suspected yet OXPHOS activities in biopsied tissue samples appear normal. We used whole exome sequencing (WES) to identify the molecular basis of an early-onset mitochondrial syndrome—pathogenic biallelic variants in the *HTRA2* gene, encoding a mitochondria-localised serine protease—in five subjects from two unrelated families characterised by seizures, neutropenia, hypotonia and cardio-respiratory problems. A unifying feature in all affected children was 3-methylglutaconic aciduria (3-MGA-uria), a common biochemical marker observed in some patients with mitochondrial dysfunction. Although functional studies of HTRA2 subjects’ fibroblasts and skeletal muscle homogenates showed severely decreased levels of mutant HTRA2 protein, the structural subunits and complexes of the mitochondrial respiratory chain appeared normal. We did detect a profound defect in OPA1 processing in HTRA2-deficient fibroblasts, suggesting a role for HTRA2 in the regulation of mitochondrial dynamics and OPA1 proteolysis. In addition, investigated subject fibroblasts were more susceptible to apoptotic insults. Our data support recent studies that described important functions for HTRA2 in programmed cell death and confirm that patients with genetically-unresolved 3-MGA-uria should be screened by WES with pathogenic variants in the *HTRA2* gene prioritised for further analysis.

**Electronic supplementary material:**

The online version of this article (doi:10.1007/s10545-016-9977-2) contains supplementary material, which is available to authorized users.

## Introduction

Mitochondrial disorders can arise at any stage of life, often presenting with a wide spectrum of clinical manifestations. Genetically and phenotypically, mitochondrial diseases collectively represent one of the most heterogeneous and common inherited groups of metabolic disorders. Due to the challenges of assessing and definitively assigning genotype-phenotype correlations in individuals, strategies to clinically and molecularly characterise patients are continually developing to provide a comprehensive molecular portfolio of bona fide variants causing mitochondrial disease. In recent years, next-generation sequencing (NGS) technologies have proven remarkably successful in identifying mutations in genes causing primary mitochondrial disorders, with more than 250 nuclear-encoded genes associated with mitochondrial syndromes identified to date (Mayr et al 2015). However, given the size of the human mitochondrial proteome—comprising ∼1300 gene products necessary to maintain mitochondrial function—many pathogenic candidate genes remain uncharacterised. This is highlighted by the many patients with mitochondrial disease still requiring a diagnosis, where the defective gene has not yet been established.

3-methylglutaconic aciduria (3-MGA-uria) is a biochemical marker of mitochondrial dysfunction in the presence of suggestive clinical features (Wortmann et al 2013a, b). Isolated 3-MGA-uria represents a clinically and genetically heterogeneous group of metabolic disorders. Based on the pathological mechanism, 3-MGA-uria syndromes have been classified into two groups, ‘primary 3-MGA-uria’ associated with defects in leucine catabolism and ‘secondary 3-MGA-uria(s)’ that are not related to leucine degradation pathways (Wortmann et al 2013a, b). The secondary 3-MGA-uria syndromes may be distinguished into three different subtypes based on the underlying pathomechanism: (1) defective phospholipid synthesis and remodelling (SERAC1 defect or MEGDEL syndrome, TAZ defect or Barth syndrome and AGK defect or Sengers syndrome) (Barth et al 1983, 2004; Kelley et al 1991; Mayr et al 2012; Wortmann et al 2012); (2) mitochondrial membrane associated diseases (OPA3 defect or Costeff syndrome, DNAJC19 defect or DCMA syndrome and TMEM70 defect) (Anikster et al 2001; Davey et al 2006; Cizkova et al 2008; Magner et al 2015) and (3) other mitochondrial proteins with unknown pathomechanism (CLPB defect) (Kanabus et al 2015; Wortmann et al 2015)

Recently, Mandel and colleagues (Mandel et al 2016) identified the first case of recessive variants in the *HTRA2* gene in four infants from two unrelated families. *HTRA2* encodes a mitochondrial-localised serine protease and its absence has been associated with 3-MGA-uria, infantile neurodegeneration, abnormal mitochondria and increased sensitivity to apoptosis (Mandel et al 2016). The HTRA2 defect represents a novel cause of inborn error of metabolism with 3-MGA-uria as a discriminative feature with an unknown pathomechanism. Here we report two additional unrelated, consanguineous families in which the affected children carried novel bi-allelic pathogenic variants in the *HTRA2* gene, leading to 3-MGA-uria, seizures, hypotonia, neutropenia and cardio-respiratory difficulties.

## Patients and methods

### Family 1 (subject 1)

**Fig. 1:**
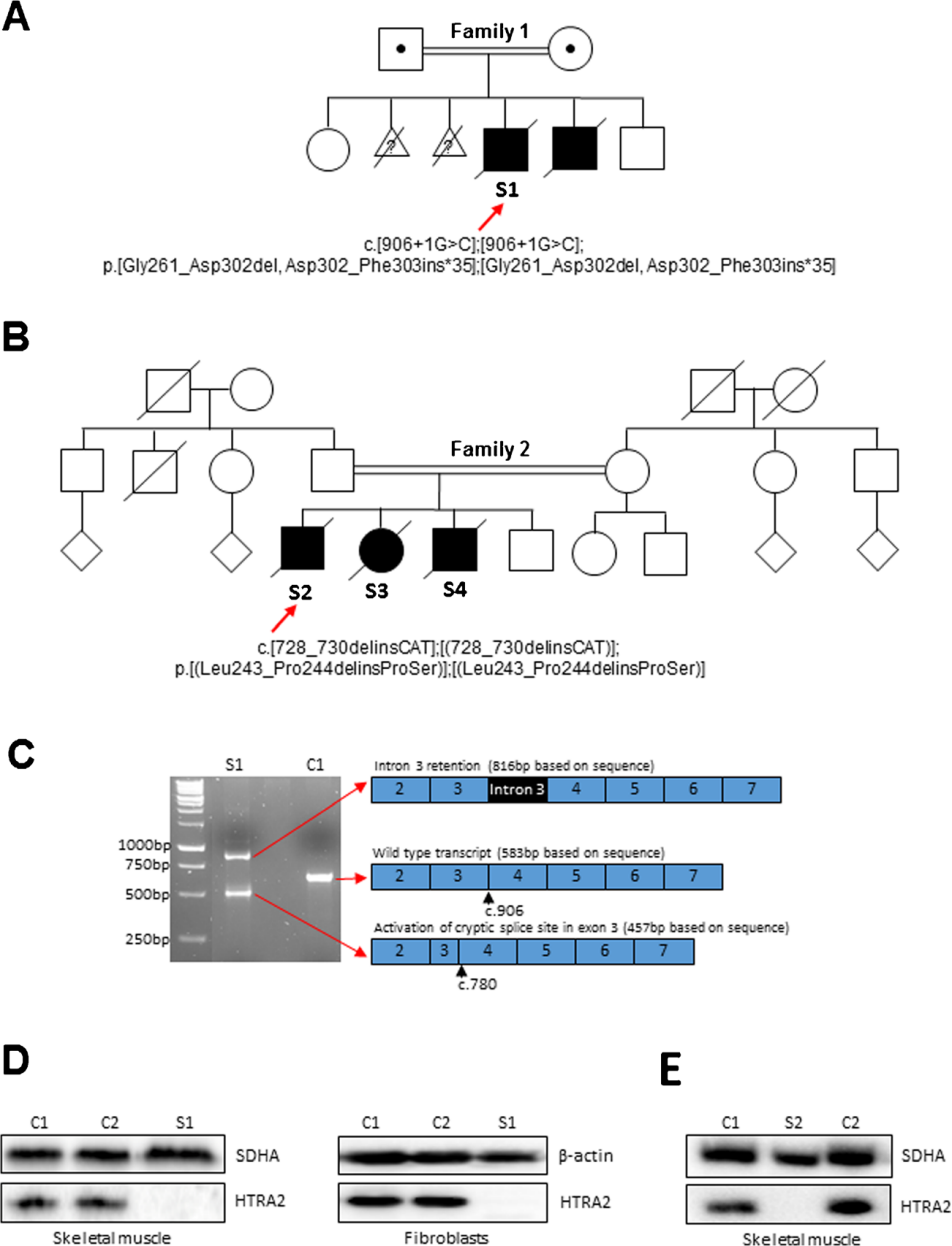
Molecular genetics and biochemical studies of *HTRA2* variants. **a**, **b** Pedigrees of the two affected families with variants in the *HTRA2* gene. The probands S1 (family 1) and S2 (family 2) are indicated by a red arrow and filled symbols denote affected individuals. **c** Amplification of cDNA across *HTRA2* exons 2–5 showed an aberrant splicing pattern for *HTRA2* RNA transcripts in S1 as two abnormal splice products were detected compared to the wild type control (C1). **d**, **e** Western blot analysis of HTRA2 levels in protein lysates isolated from control (C1, C2) and *HTRA2* S1 skeletal muscle and fibroblasts and from *HTRA2* S2 mitochondrial muscle homogenate. β-actin and SDHA antibodies were used as loading controls

The proband subject 1 (hereafter S1) was the second (male) child of first cousin Pakistani parents who also have a healthy daughter (Fig. 1a). He was born at term following an uncomplicated pregnancy. Hypoglycaemia on day 2 resolved once feeding was established and he did not require any further investigations. He was discharged aged 6 days but readmitted after 1 week with poor feeding, apneas and excessive weight loss. He was treated with anti-reflux medication and discharged, but returned 2 weeks later with recurrent apneas that progressed to central respiratory failure. On examination at 3 weeks of age, he had bilateral cataracts, hypotonia, dysphagia and dystonic limb movements. Echocardiogram showed poor cardiac contractility. The child had a respiratory arrest at 2 months of age and could not be resuscitated. In the family history, there are at least two other individuals who died in early infancy with similar medical problems as S1 but the details are not available.

Investigations showed normal liver function tests, ammonia 39 umol/L (normal range, 0–50 umol/L), CK 93 IU/L (normal range, 0–229 IU/L), LDH 379 IU/L (normal range, 240–480 IU/L) and a normal plasma amino acid profile. The urine organic acid profile showed increased excretion of 3-methylglutaconic acid (levels not quantified) with normal excretion of 3-hydroxyisovalerate on three separate occasions. The child had a persistent neutropenia (1.1×10^9^; normal range, 2.0–6.0 ×10^9^). Urine reducing substances was negative and GAL-1-PUT activity was normal. Lactate levels in the serum (1.1 mmol/L; normal range, 0.7–2.1 mmol/L) and CSF lactate (1.93 mmol/L; normal range, <2.0 mmol/L) were normal. Cranial MRI at 3 weeks of age was normal with appropriate myelination for a neonate and no abnormalities of the basal ganglia were detected.

His male sibling was born a year later at term following a normal pregnancy. He developed breathing difficulties at 3 days of age and similar feeding problems. He was fed via a nasogastric tube initially, but discontinued as the respiratory difficulties increased. He followed a similar clinical course to his brother and the parents declined further investigations and aggressive intensive care. He passed away at 3 weeks of age.

### Family 2 (subject 2)

The proband, subject 2 (hereafter S2) was a male infant born to first cousin Mexican parents at 41 weeks gestation following a normal pregnancy. Mother had two healthy children from a previous relationship (Fig. 1b). He was a large baby weighing 4360 g at the 97.6 percentile line. Vaginal vertex delivery was complicated by shoulder dystocia resulting in a left Erb’s palsy and passage of fresh meconium, some of which was aspirated. Apgar scores were poor 1^1^, 1^5^, 2^10^ and blood gas analysis revealed a pH of 7.14. Respiratory effort was weak, requiring intubation and artificial ventilation before transfer to a neonatal intensive care unit. Extubation on day 3 was unsuccessful due to hypercarbia and he required continued ventilator support for central hypopnea. He was unable to tolerate gastric feeds and received nutrition via a nasojejunal tube. Seizures began on day 5 and continued despite anticonvulsant medication. Cranial MRI revealed a widespread immaturity of cortical development with elevated lactate and lipid peaks on MRS. Skeletal muscle biopsy, performed at 2 weeks, showed muscle denervation and atrophy. Echocardiogram demonstrated a large patent ductus arteriosus. Supportive care was withdrawn after 3 months when he continued to demonstrate respiratory insufficiency and he died soon thereafter. Post mortem examination was declined. Metabolic investigation revealed persistent elevation of 3-methylglutaric and 3-methylglutaconic acids in urine. Additional metabolic studies showing normal results included plasma and urinary creatine and guanidinoacetate and plasma thymidine. Diagnostic genetic studies showed no known deleterious variants in *TAZ*, *TMEM70*, *POLG*, *SUCLA2*, *SUCLG1* or in mtDNA.

The second affected infant in this family, subject 3 (S3), is the sister of the index case who was born by vaginal delivery at term weighing 3829 g with Apgar scores of 1^1^, 1^5^ and 8^10^. Although attempts at breast and bottle-feeding were noted to be poor she was discharged from hospital on day 3, only to be readmitted on day 7 with jaundice that required 2 days of treatment. She was admitted again on day 27 with poor feeding, weight loss and suspected seizures. Respiratory function rapidly deteriorated on admission requiring intubation and artificial ventilation for a period of 2 months, after which time she was discharged, only to be readmitted 2 days later with seizure-like activity and respiratory failure. Palliative therapy was offered at home and she died 2 days later aged 4 months. A post mortem was declined. Urine organic acid analysis showed elevated 3-methylglutaric acid and 3-methylglutaconic acid.

The third affected child in this family, subject 4 (S4), was a male born at 39 weeks gestation by elective caesarean section for breech presentation. Apgar scores were good (7, 9) and the immediate postnatal period uneventful. However, on day 2, he developed profound bradycardia, tremors and clonus of both lower extremities. Urine organic acid analysis confirmed 3-methylglutaric and 3-methylglutaconic acids and he was rapidly transitioned to a palliative care pathway without further investigation. He died at home at the age of 3 months. Post mortem was not performed.

### Whole exome sequencing analysis

Whole exome sequencing (WES) was undertaken for S1 and S4 probands (Illumina HiSeq 2500 or Illumina HiSeq 2000) using previously described methodologies and bioinformatics filtering pipelines (Jones et al 2012; Bonnen et al 2013; Besse et al 2015). Clinical WES was conducted on S4 at the Baylor Miraca Genetics Laboratory. Putative pathogenic variants were confirmed by Sanger sequencing of PCR-amplified products using BigDye Terminator cycle sequencing chemistry (Applied Biosystems, ABI) on an ABI3130xl Genetic Analyser. Sequence data were analysed using Mutation Surveyor software v4.0.9 (SoftGenetics). Variant nomenclature was annotated in reference to GenBank NM_013247.4 according to Human Genome Variation Society (HGVS) guidelines.

### cDNA studies

Total RNA was extracted from fibroblasts cultured from S1 using the ReliaPrep RNA Cell Miniprep System (Promega) and reverse-transcribed to cDNA using the GoScript Reverse Transcription System (Promega), according to the manufacturer’s guidelines. The cDNA was then amplified across exons 2–5 using exonic primers (primer sequences and PCR conditions available upon request) and PCR products were separated by agarose gel electrophoresis using standard conditions. PCR products were directly sequenced as described above.

### Cell culture conditions

Primary paediatric control and *HTRA2* subjects’ fibroblasts were cultured in Dulbecco’s modified Eagle’s medium (Sigma) supplemented with 10 % fetal calf serum, 1 x non-essential amino acids and 50 μg/ml uridine. Cell lines were propagated at 37 °C in humidified 5 % CO_2_.

### Western blot and blue native analysis

Western blot and blue native analysis was performed on available fibroblast and skeletal muscle protein samples from the two affected subjects according to previously described methodologies (Oláhová et al 2015; Oláhová, Hardy et al 2015) and as indicated in Supplementary materials and methods.

### Mitochondrial network analyses and confocal microscopy

Control (C1, C2) and subject (S1) fibroblasts were grown in glass-bottomed wells (WillCo) to permit assessment of mitochondria and following incubation of cells at 37 °C for 45 minutes in the presence of the PicoGreen solution (Invitrogen) at 3 μl/ml, followed by a 15 minute incubation with 5nM TMRM (Invitrogen). PicoGreen was used to visualise mitochondrial nucleoids and TMRM dye that is sequestered by active mitochondria allowed the visualisation of the mitochondrial network. Cells were washed in standard growth media containing 5nM TMRM and images captured using a 63x magnification oil immersion objective on an inverted point scanning confocal microscope (Nikon A1R). Z-stack images were processed and analysed by ImageJ. Images were binarised allowing the automatic quantification of mitochondrial morphological characteristics shown as the aspect ratio (AR) measuring mitochondrial length, perimeter and area. Form factor (FF) was calculated by perimeter^2^/4pi∙area and represents mitochondrial lengths and degree of branching. Measurements were obtained from duplicate experiments (each n = 10) and compared for differences between S1 and two passage-matched controls (two-tailed unpaired Student’s t-test).

### Electron microscopy

Cultured fibroblasts were grown on Transwell^®^ polyester membrane cell culture inserts, fixed with 2 % glutaraldehyde in 0.1 M cacodylate buffer and processed using the heavy metal impregnation protocol as described previously (Deerinck et al 2010). Briefly, the cells were immersed in 3 % potassium ferrocyanide + 2 % osmium tetroxide, followed by 0.1 % thiocarbohydrazide, then 2 % osmium tetroxide and finally left overnight in 1 % uranyl acetate (with water washes between each step). The next day the cells were immersed in 0.6 % lead aspartate solution, dehydrated in graded acetone and embedded in epoxy tab 812 hard resin. After polymerisation, ultrathin sections were picked up on copper grids for transmission electron microscopy (TEM) and the rest of the resin block was used for serial block face scanning EM imaging (SBF-SEM). Mitochondrial morphology was analysed with a Zeiss SEM incorporating the 3View GATAN^®^ and a Philips CM100 TEM. Images were processed in Digital Mircograph^®^ and ImageJ.

### Apoptosis assay

Control (C1) and subject (S1) fibroblasts were treated with either 0.3 μM staurosporine or vehicle alone 0.2 % dimethyl sulfoxide (DMSO) for 8 hours. Apoptotic cells were analysed in cultured fibroblasts using the APO-Direct Kit (BD Biosciences) following manufacturer’s instructions. DNA breaks were detected by labelling cells (*n* = 1 × 10^6^) with the terminal deoxynucleotidyl transferase conjugated to FITC. Cells were exposed to excitation with 488 nm laser, following FACS analysis performed on CANTO II flow cytometer. Total DNA was stained with propidium iodine.

## Results

### Whole exome sequencing analysis identifies *HTRA2* variants

DNA from S1 (family 1) was subjected to WES, with bioinformatic filtering of the raw data undertaken in a step-wise process to prioritise genes encoding proteins with a known or predicted mitochondrial localisation harbouring rare, recessively-inherited (compound heterozygous or homozygous) variants. This facilitated the identification of pathogenic *HTRA2* variants. S1 was homozygous for a c.906 + 1G > C splice site variant, which disrupts the consensus donor splice site of intron 3 and leads to a complex, aberrant splicing pattern of *HTRA2* RNA transcripts (Fig. 1c and Supplementary Fig. 1). Two abnormal splice products were observed following RT-PCR: one shorter product resulting from the activation of a cryptic splice site in exon 3 (removing nucleotides r.781_906 from this transcript) and a longer product resulting from complete retention of intron 3 (Fig. 1c and Supplementary Fig. 1). The predicted effects of these splicing patterns at the protein level are an in-frame deletion of 42 amino acids (p.Gly261_Asp302del) in the shorter transcript and translational read-through of intron 3 resulting in the inclusion of 34 amino acids until a termination codon is encountered at position 35 (p.Asp302_Phe303ins*35) in the longer transcript. There was no detectable evidence of a wild type transcript using this method. Testing of parental samples confirmed carrier status. A molecular diagnosis was made in the family whilst the mother was carrying her 6th pregnancy; prenatal testing (CVB biopsy) at 12 weeks of gestation confirmed the absence of the c.[906 + 1G > C];[906 + 1G > C] *HTRA2* variant, and following a normal delivery this male child continues to develop well.

In S4 (family 2), clinical WES identified a variant in *HTRA2* c.728_730delinsCAT; p.Leu243_Pro244delinsProSer in an apparently homozygous state. Parental samples were not available for segregation analysis, but affected sibling, S2 (family 2), was shown by Sanger sequencing to be homozygous for this variant consistent with a shared clinical phenotype. Both residues, p.Leu243 and p.Pro244, show evidence of being evolutionarily constrained with PhyloP scores above 2.0, and are situated within a highly conserved peptidase domain, whilst the mutation in this family does not appear to give rise to a frameshift or nonsense allele, thereby providing a trigger for nonsense-mediated decay.

### Deleterious variants in *HTRA2* lead to complete loss of HTRA2 protein

To determine the deleterious nature of the *HTRA2* gene defect on protein level, control (C1, C2) and subject fibroblasts (S1) or skeletal muscle (S1, S2) samples were analysed by western blotting. The steady-state levels of HTRA2 in subject fibroblasts (S1) and skeletal muscle (S1 and S2) were undetectable when compared to age-matched controls (Fig. 1d and e), strongly implying that both variants affect the translation, stability and/or the turnover of the HTRA2 protein.

### OXPHOS steady-state levels and complex assembly

**Fig. 2:**
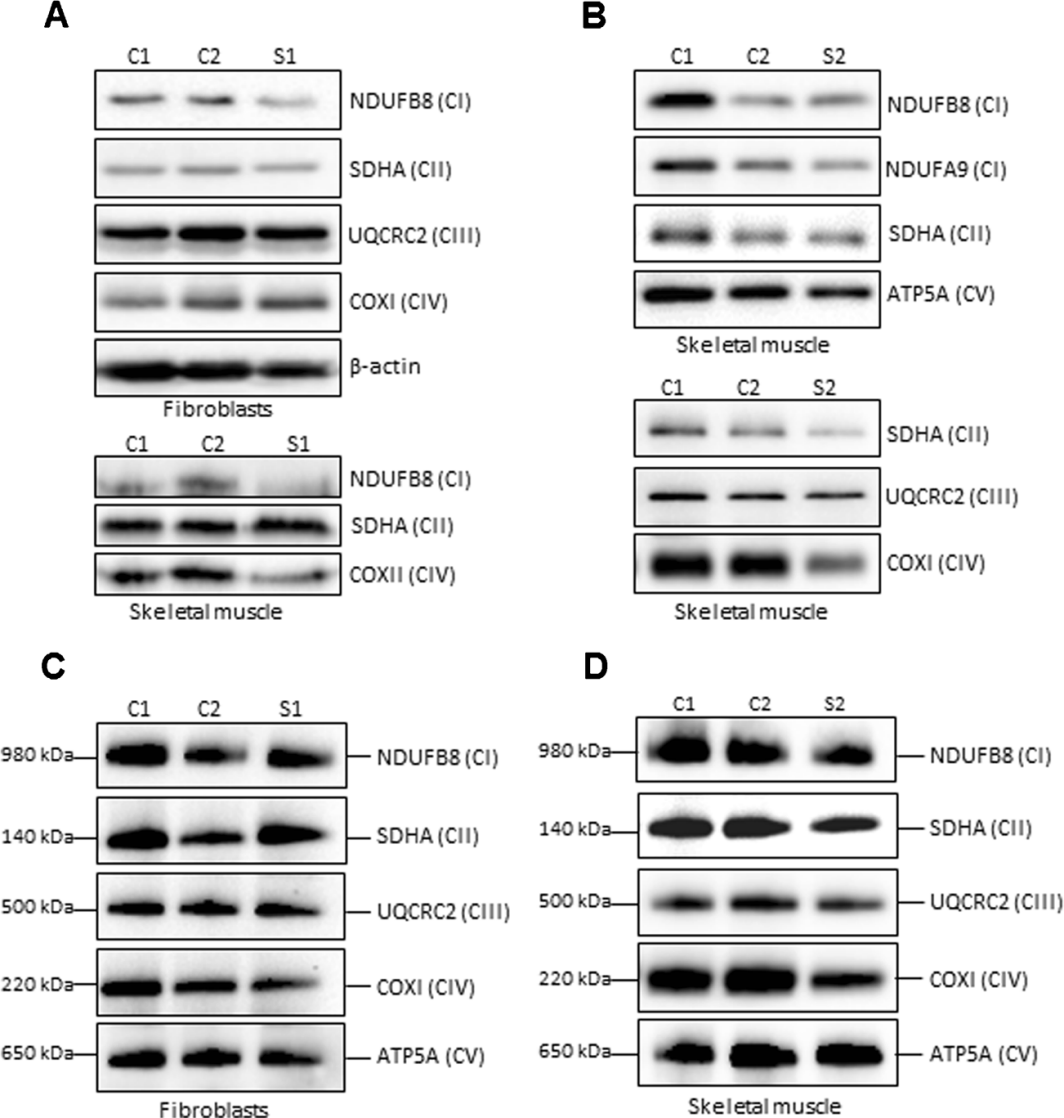
Steady-state levels and assembly of OXPHOS complexes. **a**, **b** SDS-PAGE (12 %) and immunoblot analysis of steady-state levels of OXPHOS complex subunits in protein extracts (30ug) isolated from age-matched control (C1, C2) and **a** *HTRA2* S1 fibroblasts (upper panel) and skeletal muscle (lower panel). **b** Mitochondrial lysates (40ug) from control and *HTRA2* subject’s (S2) skeletal muscle were used to analyse the levels of OXPHOS complex subunits. In **a** and **b** β-actin and SDHA served as loading controls. **c**, **d** One-dimensional BN-PAGE analysis of the assembly of individual OXPHOS complexes in DDM-solubilised mitochondrial extracts from control (C1, C2) and *HTRA2* subject’s **c** fibroblasts (S1) and **d** skeletal muscle (S2). Complex II (SDHA) was used as a loading control

To characterise the impact of *HTRA2* variants on the steady-state levels of mitochondrial respiratory chain complex subunits, western blot analysis was performed on available fibroblast and skeletal muscle protein samples from the two affected subjects. The loss of HTRA2 did not significantly affect the steady-state levels of investigated OXPHOS subunits (Fig. 2a and b). Protein lysates from S1 fibroblasts and skeletal muscle revealed a marginal decrease in the levels of the complex I subunit NDUFB8 (Fig. 2a). In addition, a slight decrease was detected in the levels of the mitochondrial-encoded COXII component of complex IV subunit in S1 skeletal muscle (Fig. 2a). Investigation of mitochondrial extracts from skeletal muscle tissue available from S2 revealed a slight decrease in the steady-state levels of COXI subunit of complex IV (Fig. 2b). The levels of other investigated complexes (I, II, III and V) were normal in S2 skeletal muscle (Fig. 2b). In addition, normal respiratory chain complex activities (complexes I-IV) were reported following diagnostic screening in a muscle homogenate from S2 (data not shown). The nuclear-encoded subunit of complex II, SDHA, was used as a loading control.

One-dimensional blue native-PAGE (BN-PAGE) analysis was performed to interrogate the assembly of mitochondrial OXPHOS complexes in *HTRA2* subjects. Due to limited availability of muscle tissue from S1, the OXPHOS complex assembly was determined only in fibroblasts. We observed a minor defect in complex IV assembly in S1 fibroblasts and S2 muscle samples (Fig. 2c and d). The levels of fully assembled complexes I, II, III and V were comparable to the control fibroblasts and muscle tissue controls (C1, C2), respectively (Fig. 2c and d). The nuclear-encoded subunit of complex II, SDHA, was used as a loading control.

### Differential OPA1 proteolysis in *HTRA2* subjects

**Fig. 3:**
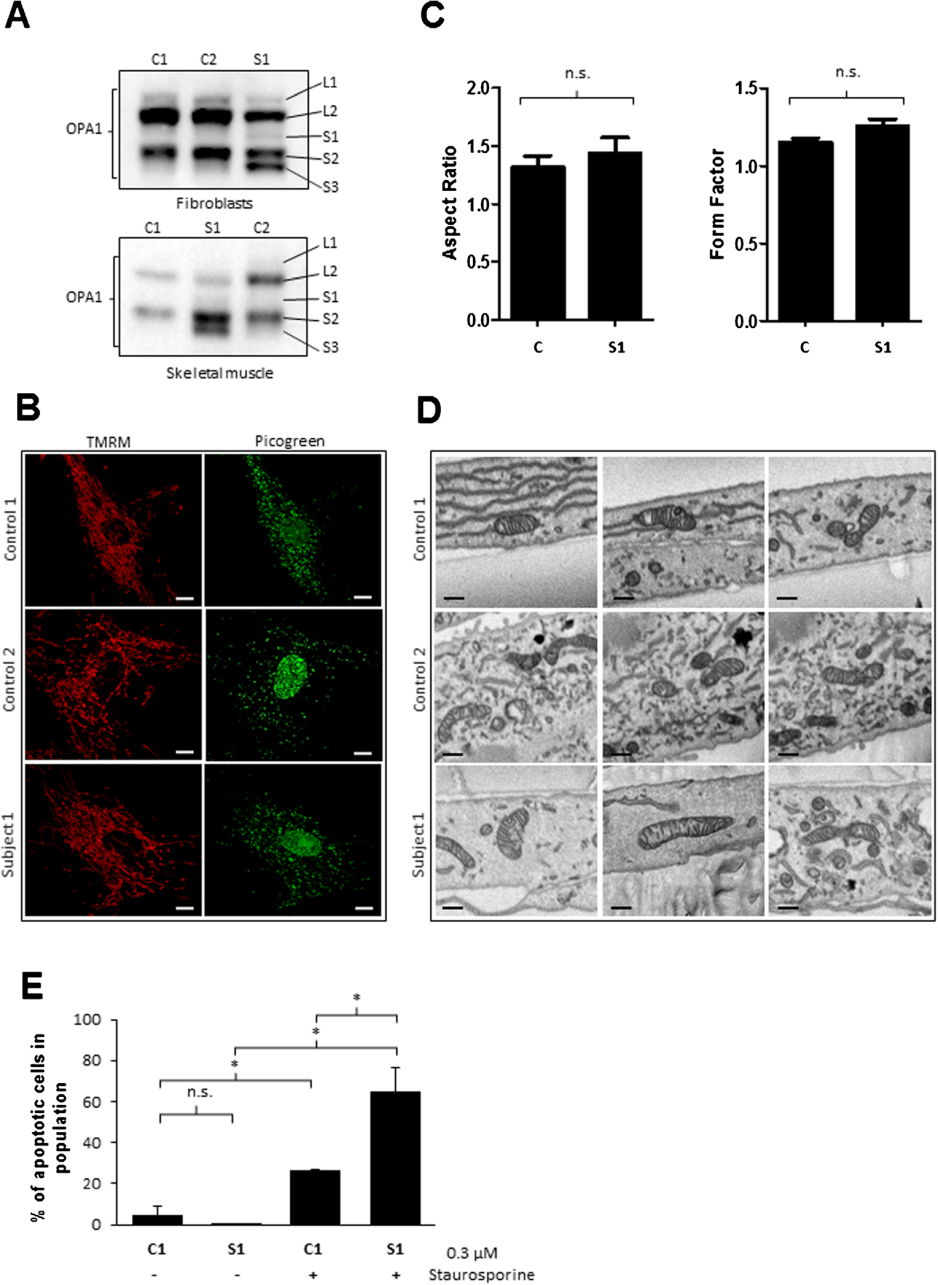
Analysis of mitochondrial morphogenesis and apoptosis. **a** The effects of loss of HTRA on OPA1 protein levels in control (C1, C2) and subject (S1) fibroblasts and skeletal muscle were analysed by SDS-PAGE (7 %) and immunoblotting against the mitochondrial fusion protein OPA1. Differences in the OPA1 proteolytic cleavage pattern between control and subject samples were detected, with increased levels of short OPA1 cleavage products present in the *HTRA2* subject (S1-S3). **b** The panels on the left show representative images of TMRM staining in control C1 (upper), control C2 (middle) and subject S1 (lower) fibroblasts, revealing a well-connected tubular mitochondrial network in subject 1 consistent with both controls. The panels on the right show representative images of nucleoid staining by PicoGreen in control C1 (upper), control C2 (middle) and subject S1 (lower) fibroblasts. Nucleoid size and distribution in S1 is comparable to both controls. Scale bar = 10 μm. **c** Quantitative analysis of mitochondrial network demonstrated a non-significant difference in aspect ratio (left panel) and form factor (right panel) of S1 compared to the controls. All data shown are represented as mean ± SEM from two independent experiments. Statistical analysis was performed using a two-tailed unpaired Student’s t-test, ns = not significant. **d** Representative SBF-SEM images of mitochondria from control (C1, C2) and *HTRA2* subject (S1) fibroblasts showing morphologically normal mitochondria with no obvious defects in cristae structure. Scale bar = 1 μm. **e** Quantitative analysis of the percentage of apoptotic cells in population (*n* = 10 000) in control (C1) and subject (S1) fibroblasts treated with or without 0.3 μM Staurosporine (ST) for 8 h. Results are shown as mean STDEV (*n* = 2); paired Student’s t-test: C1 vs S1 *p* = n.s., C1 vs C1 + ST *p* = 0.01; S1 vs S1 + ST *p* = 0.01 and C1 + ST vs S1 + ST *p* = 0.03

Mitochondrial dynamics are regulated by a number of well-characterised, inner mitochondrial membrane proteins that facilitate continuous fission and fusion of mitochondria. A key player in this process is OPA1, a dynamin-related GTPase that regulates mitochondrial fusion, cristae structure and apoptosis (Olichon et al 2003; Frezza et al 2006). The balance between the levels of long-OPA1 (L-OPA1) and short-OPA1 (S-OPA1) cleavage products plays an important role in the fusion and fission processes, and previous studies have reported a physical interaction between HTRA2 and OPA1 in mammals (Kieper et al 2010). Here, we examined the levels of OPA1 in the HTRA2-deficient tissues and found differential proteolytic processing of OPA1. In both, skin fibroblasts and skeletal muscle derived from S1, we detected increased amounts of S-OPA1 cleavage products (Fig. 3a). The increased proteolytic processing of the L-OPA1 forms into S-OPA1 products in the subject samples suggest that HTRA2 may affect mitochondrial fusion and OPA1 proteolysis.

### Analysis of mitochondrial network and morphology

In order to further investigate the effect of *HTRA2* variants on mitochondrial dynamics, we assessed the mitochondrial network and distribution of mitochondrial nucleoids in control and S1 fibroblasts (Fig. 3b and c). The distribution and size of the nucleoids appeared normal in subject fibroblasts when compared to both controls (Fig. 3b and c). Similarly, mitochondria in subject fibroblasts displayed a well-connected tubular network that was comparable to the control cell lines (Fig. 3b and c).

Serial block face SEM of S1 fibroblasts did not reveal any marked abnormalities in mitochondrial morphology and cristae structure when compared to controls (Fig. 3d). Transmission EM suggested that a subset of mitochondria in S1 fibroblasts exhibited a decreased amount of inner mitochondrial membrane structures, however, these changes were also observed to a similar level in both sets of control fibroblasts (Supplementary Fig. 2). These results indicate that, although certain changes have been observed, the absence of HTRA2 does not have an immense impact on mitochondrial network and ultrastructure.

### Investigation of apoptosis in *HTRA2* subject fibroblasts

HTRA2 has been reported to play an important role in the regulation of programmed cell death (Suzuki et al 2001; Hegde et al 2002; Martins et al 2002; Kang et al 2013; Mandel et al 2016). The release of HTRA2 into the cytosol has been linked with both caspase-dependent and caspase-independent activation of apoptosis (Vande Walle et al 2008). To investigate the effect of loss of HTRA2 on apoptosis, studied subject fibroblasts (S1) were treated in the presence of an apoptosis-inducing agent, staurosporine. Following 8 h treatment in the presence of 0.3 μM staurosporine, the cellular morphology of both, control and subject fibroblasts was altered compared to cells treated with vehicle alone DMSO. Staurosporine exposure induced cellular shrinkage and spherical cell bodies with thin stellate-like projections, whilst cells treated with DMSO alone had a typical flattened appearance (data not shown). Quantitative flow cytometry analysis of apoptotic cells in the absence of staurosporine did not show any significant increase in apoptosis in both control and subject fibroblasts (Fig. 3e and Supplementary Fig. 3). Conversely, staurosporine treatment increased the sensitivity of HTRA2 deficient fibroblasts to apoptotic insults (2.5 fold) compared to control cells (Fig. 3e and Supplementary Fig. 3). These data suggest that in the absence of HTRA2, cells are more susceptible to apoptosis, thus further supporting the purported role of HTRA2 in the regulation of apoptotic pathways.

## Discussion

Using WES, we identified the molecular basis of increased 3-MGA-uria in five individuals from two unrelated families presenting with a variety of phenotypes including neutropenia, hypotonia, dystonia, seizures, bilateral cataracts, central apneas, feeding difficulties and cardio-respiratory symptoms. The underlying genetic defect in both families was due to homozygous variants in the *HTRA2* gene, c.906 + 1G > C; p.(Gly261_Asp302del,Asp302_Phe303ins*35) and c.728_730delinsCAT; p.(Leu243_Pro244delinsProSer), respectively.

In adults, variants in *HTRA2* have been associated with development of tremor and Parkinson’s disease (PD) (Unal Gulsuner et al 2014; Tzoulis et al 2015). Despite the identification of a large number of patients carrying PD *HTRA2* variants, the exact role of HTRA2 in PD still remains controversial (Kruger et al 2011). Recent studies have identified the first case of recessively-inherited pathogenic variants in the *HTRA2* gene [c.1211 G > A; p.(Arg404Gln) and c.1312_1316del; p.(Ala438fs)] in four infants from two unrelated families (Mandel et al 2016). In many aspects, the clinical features of the individuals reported here very closely resemble those in the recent publication (Mandel et al 2016). For example, affected children presented with dysphagia and recurrent apnea, hypotonia, bradycardia and seizures, whilst increased 3-MGA-uria was a prominent feature in all four subjects.

*HTRA2* encodes an ATP-independent serine protease that resides in the mitochondrial intermembrane space, where it acts in a protein quality control system. To date a number of mitochondrial disease causing genes involved in mitochondrial protein quality control have been identified. Among these are the heat shock protein/chaperonin CLPB (Kanabus et al 2015; Wortmann, Zietkiewicz et al 2015), the mitochondrial matrix peptidase CLPP (Jenkinson et al 2013), subunits of the mitochondrial matrix m-AAA protease AFG3L2 and SPG7 (Cagnoli et al 2010; Pfeffer et al 2014) and the conserved heat shock protein 60 HSPD1 (Hansen et al 2007). Interestingly, patients harbouring variants in CLPB protease are characterised by increased 3-MGA-uria, neutropenia, epilepsy, cataracts and movement disorders, which are some of the prominent features found in the individuals presented here (Kanabus et al 2015; Wortmann et al 2015). It is possible that the protease activity of HTRA2 could contribute to certain post-transcriptional modifications of proteins that are directly associated with inborn errors of metabolism with 3-MGA-uria as discriminative feature.

The primary function of HTRA2 in mitochondria is to maintain mitochondrial protein homeostasis. However, studies have also reported that upon apoptotic stimuli, HTRA2 is recruited to the cytosol where it activates apoptotic caspases (Quiros et al 2015). Indeed, our results suggest that complete loss of HTRA2 in the studied subject (S1) fibroblasts leads to increased sensitivity to apoptosis (Fig. 3e and Supplementary Fig. 3). These findings are in agreement with recent studies showing increased apoptotic susceptibility in patients carrying recessive variants in *HTRA2* (Mandel et al 2016) and a mouse model lacking neuronal *HTRA2* (Kang et al 2013).

Human *HTRA2* variants had a mild, or no effect, on the steady state levels and assembly of mitochondrial respiratory chain complexes in the fibroblasts and skeletal muscle homogenates studied here. Our findings show that loss of HTRA2 influences the proteolytic processing of the mitochondrial dynamics regulator OPA1. The ubiquitously expressed OPA1 is processed into L- and S-OPA1 protein products, each of which have a distinct role in the control of mitochondrial fission and fusion processes, cristae morphology and apoptosis (MacVicar and Langer 2016). Mutated HTRA2 caused differential proteolytic cleavage of OPA1 resulting in a marked increase of S-OPA1 forms that have been associated with fragmented mitochondria. Despite the increased amounts of S-OPA1 cleavage products detected in *HTRA2* subjects’ fibroblasts, we did not observe any significant changes in the mitochondrial network or ultrastructure. Although the exact role of HTRA2 in the regulation of mitochondrial morphology is not clear and it requires further investigation, it can be hypothesised that HTRA2 indirectly mediates OPA1 cleavage, initiating a different mode of mitochondrial fission-fusion represented by transient fusion that is influenced by differentially expressed OPA1 levels (Liu et al 2009). During transient fusion events mitochondria rapidly exchange soluble contents whilst maintaining normal mitochondrial network (Liu et al 2009)—a mode observed in the fibroblasts derived from the individual carrying deleterious *HTRA2* variants.

In conclusion, we report the identification of deleterious, biallelic variants in the *HTRA2* gene encoding a mitochondria-localised serine protease. *HTRA2* variants represent a novel and significant cause of rapidly progressing, neonatal onset mitochondrial disease associated with 3-MGA-uria, hypotonia, dystonia, seizures and cardio-respiratory difficulties. WES is proving to be a powerful tool for investigating the genetic cause of mitochondrial syndromes in individuals with clinical evidence of mitochondrial disease, but no other supportive biochemical or genetic findings. Our findings confirm *HTRA2* to be an important candidate disease gene that should either be directly screened in patients with increased levels of 3-MGA, or variants in this gene identified by WES prioritised for further analysis.

## Electronic supplementary material

Below is the link to the electronic supplementary material.

ESM 1 (DOCX 18 kb)

Supplementary Fig. 1 The *HTRA2* coding region spanning across exons 2–5 was amplified and sequenced. Sanger sequencing analysis revealed that the c.906 + 1G > C variant produces two abnormal splicing products by i) removing nucleotides r.781_906 and ii) producing a longer product by complete retention of intron 3. (GIF 228 kb)

High resolution image (TIF 195 kb)

Supplementary Fig. 2 Representative images of mitochondria from control 1 (C1) [A-D], control 2 (C2) [E-H] and *HTRA2* subject’s (S1) [I-L] fibroblasts visualised by TEM. Examples of mitochondria with loss of inner mitochondrial membrane ultrastructure in both, control and subject fibroblasts (indicated by a black arrow). (GIF 564 kb)

High resolution image (TIF 1031 kb)

Supplementary Fig. 3 FACS analysis of apoptotic cells in control (C1) and subject (S1) fibroblasts treated without [A-B] or with [C-D] 0.3 μM Staurosporine for 8 h using the APO-DIRECT Kit. Representative FACS data for each group (*n* = 10 000 cells) are shown. Non-apoptotic cells are shown in green and the increased FITC fluorescence signal indicates the presence of apoptotic cells shown in pink. (GIF 200 kb)

High resolution image (TIF 163 kb)
