# Supplementary material for: Pathogenic variants in HTRA2 cause an early-onset mitochondrial syndrome associated with 3-methylglutaconic aciduria
Source: J Inherit Metab Dis. 2016 Sep 30;40(1):121–30. doi: 10.1007/s10545-016-9977-2 (PMC5203855; doi:10.1007/s10545-016-9977-2)
Supplement: Supplementary file 1 — (DOCX 18 kb) [file 10545_2016_9977_MOESM1_ESM.docx]

**SUPPLEMENTARY DATA**

**Supplementary materials and methods:**

**Cell lysis and Western blot analysis**

Cultured fibroblasts were harvested and lysed for 30 minutes on ice in lysis buffer containing

# 50mM Tris-HCl pH 7.5, 130mM NaCl, 2mM MgCl_2_, 1mM  phenylmethylsulfonyl fluoride  (PMSF), 1% Nonidet P-40 (v/v) and 1 x EDTA free protease inhibitor cocktail (Pierce). Muscle homogenates were powdered in liquid nitrogen using pestle and mortar. 1ml of RIPA buffer (1% Igepal, 1.5% Triton X-100, 0.5% sodium deoxycholate, 0.1% sodium dodecyl sulphate (SDS), 10mM β-mercaptoethanol, 1mM PMSF and 1 x EDTA free protease inhibitor cocktail (Pierce) was added to powdered tissue and incubated on ice for 45 minutes. Following 2 x 15 seconds homogenization with the polytron homogenizer, protein lysates were cleared by centrifugation at 14 000*g* for 10 minutes at 4°C. Protein concentration was determined by Bradford assay (Bio-Rad). 30-40 µg of each protein lysate was separated by SDS polyacrylamide electrophoresis (SDS PAGE), followed by a transfer to PVDF membranes. Immunoblot analysis was performed using primary HTRA2 (AF1458, R&D Systems), β-actin (A1978, Sigma), OPA1 (612606, BD Transduction Laboratories) and OXPHOS complex-specific antibodies [NDUFB8 (ab110242, Abcam), NDUFA9 (MS111, Molecular Probes), SDHA (459200, MitoSciences), UQCRC2 (ab14745, Abcam), COX1 (ab14705, Abcam), COX2 (ab110258, Abcam), ATP5A (ab14748, Abcam)] and appropriate HRP-conjugated secondary antibodies (DAKO, PO399 and P0260).

**Mitochondrial preparation and Blue Native electrophoresis**

Mitochondria from skin fibroblasts and skeletal muscle were prepared exactly as previously described in ([Olahova, Haack et al. 2015](file:///D:\Programs\ProductionJournal\Temp\10545_9977.doc#_ENREF_29); [Olahova, Hardy et al. 2015](file:///D:\Programs\ProductionJournal\Temp\10545_9977.doc#_ENREF_31)). Briefly, cultured fibroblasts were homogenized in 0.6M mannitol, 1mM EGTA, 10mM Tris-HCl pH 7.4, 1mM PMSF and 0.1 % (v/v) BSA using a Teflon glass Dounce homogenizer (3 x 15 strokes). Standard differential centrifugation was used to isolate mitochondria. Similarly, muscle tissue was homogenised in 250mM sucrose, 20mM Imidazole-HCl pH 7.4 and 1mM PMSF and subjected to 1 x 20 passes of homogenization. Following centrifugation at 20 000*g* for 10 minutes at 4 °C, the pellet was washed twice in the homogenisation buffer described above, before solubilisation with n-dodecyl β-D-maltoside (DDM; Sigma). Samples of DDM solubilised mitochondrial extracts (100µg) were separated in the first-dimension on 4-16% native polyacrylamide BisTris gel (Life Technologies) before electrophoretic transfer to PVDF membranes and being subjected to immunoblotting using OXPHOS specific antibodies.
