# Supplementary figures and images for: Pathogenic variants in HTRA2 cause an early-onset mitochondrial syndrome associated with 3-methylglutaconic aciduria
Source: J Inherit Metab Dis. 2016 Sep 30;40(1):121–30. doi: 10.1007/s10545-016-9977-2 (PMC5203855; doi:10.1007/s10545-016-9977-2)

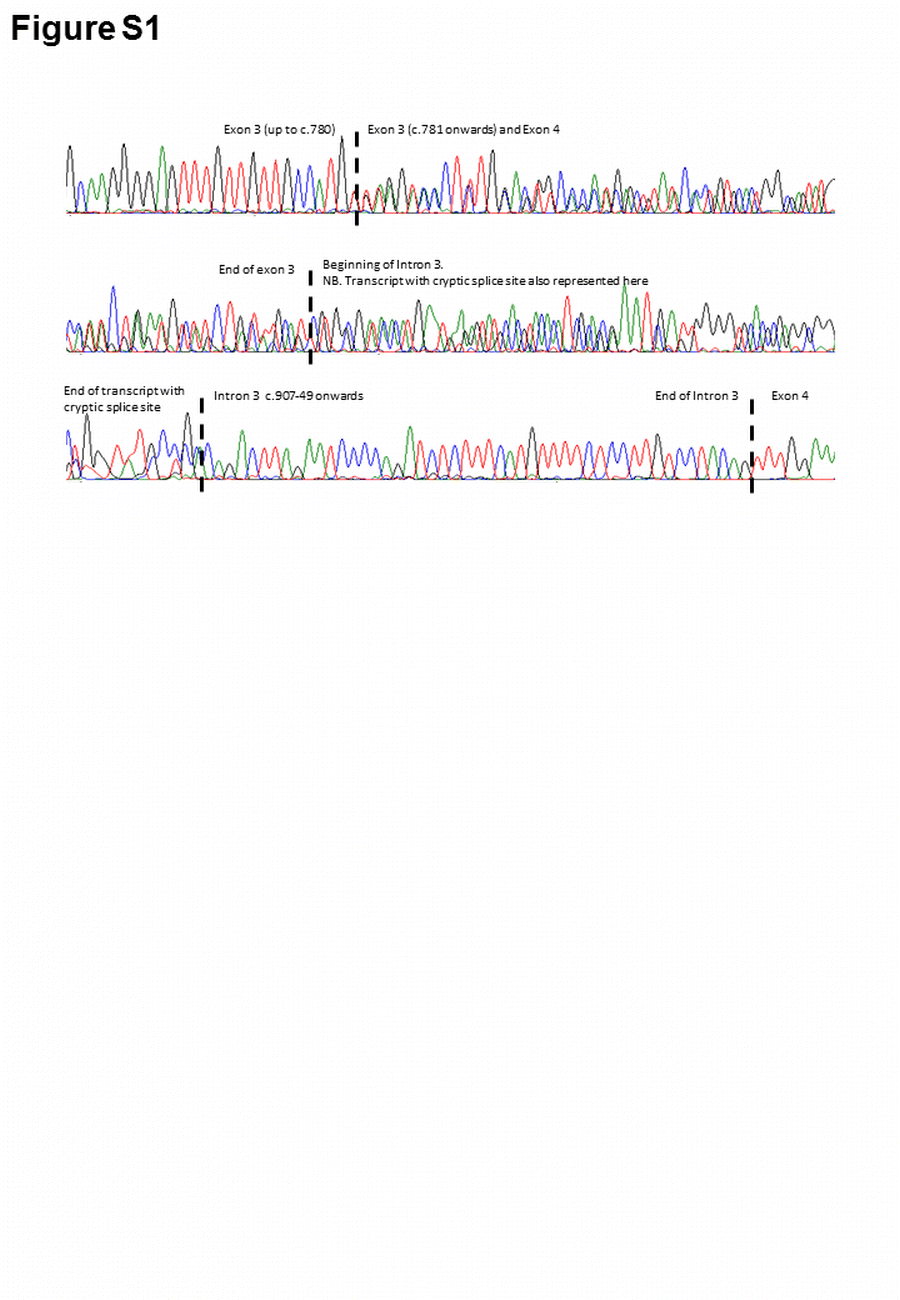

Supplement: Supplementary file 2 — The HTRA2 coding region spanning across exons 2–5 was amplified and sequenced. Sanger sequencing analysis revealed that the c.906 + 1G > C variant produces two abnormal splicing products by i) removing nucleotides r.781_906 and ii) producing a longer product by complete retention of intron 3. (GIF 228 kb) [file 10545_2016_9977_Fig4_ESM.gif]

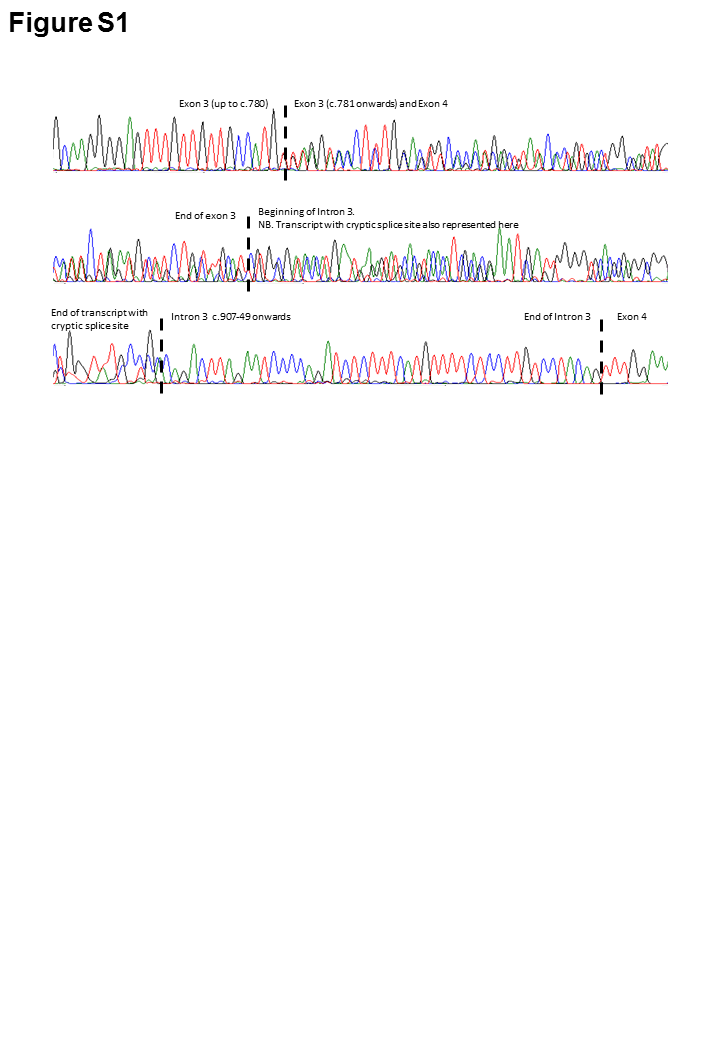

Supplement: Supplementary file 3 — High resolution image (TIF 195 kb) [file 10545_2016_9977_MOESM2_ESM.tif]

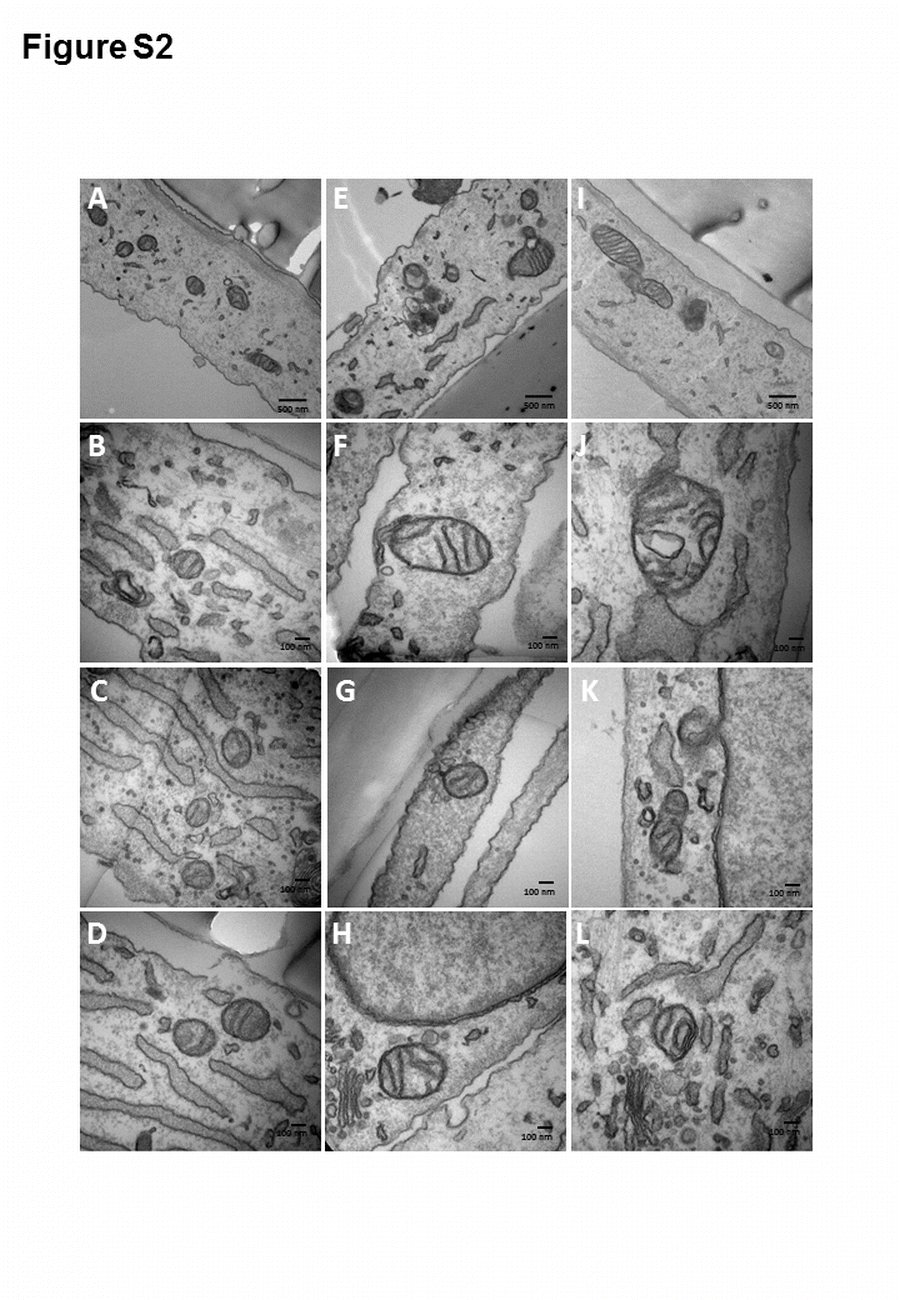

Supplement: Supplementary file 4 — Representative images of mitochondria from control 1 (C1) [A-D], control 2 (C2) [E-H] and HTRA2 subject’s (S1) [I-L] fibroblasts visualised by TEM. Examples of mitochondria with loss of inner mitochondrial membrane ultrastructure in both, control and subject fibroblasts (indicated by a black arrow). (GIF 564 kb) [file 10545_2016_9977_Fig5_ESM.gif]

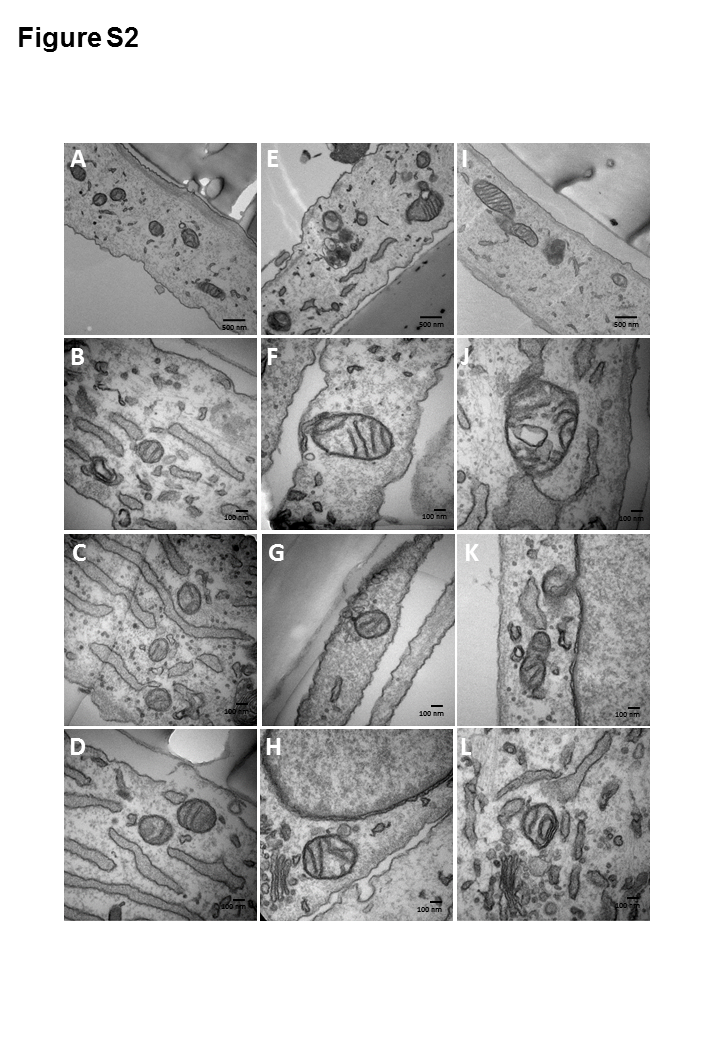

Supplement: Supplementary file 5 — High resolution image (TIF 1031 kb) [file 10545_2016_9977_MOESM3_ESM.tif]

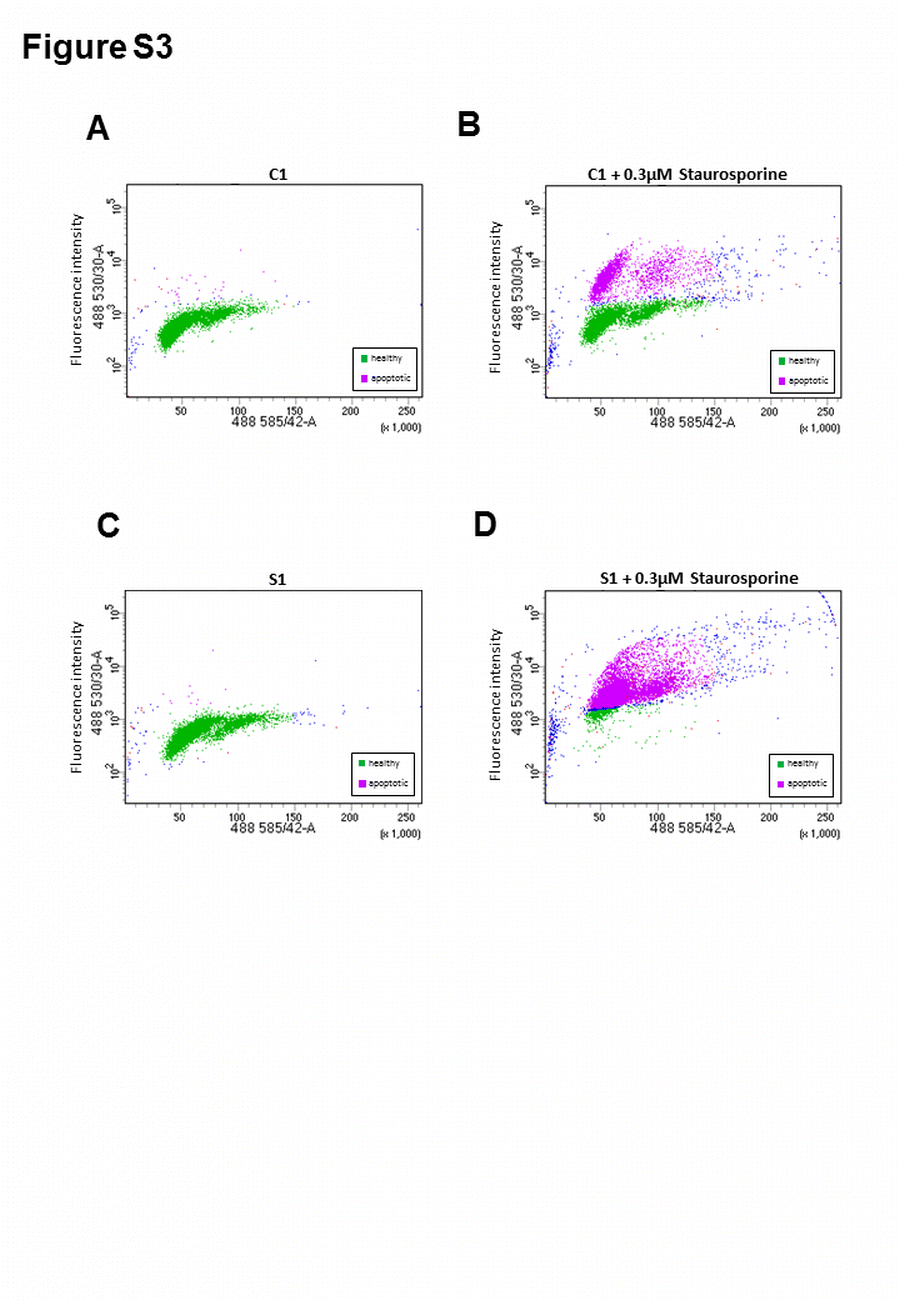

Supplement: Supplementary file 6 — FACS analysis of apoptotic cells in control (C1) and subject (S1) fibroblasts treated without [A-B] or with [C-D] 0.3 μM Staurosporine for 8 h using the APO-DIRECT Kit. Representative FACS data for each group (n = 10 000 cells) are shown. Non-apoptotic cells are shown in green and the increased FITC fluorescence signal indicates the presence of apoptotic cells shown in pink. (GIF 200 kb) [file 10545_2016_9977_Fig6_ESM.gif]

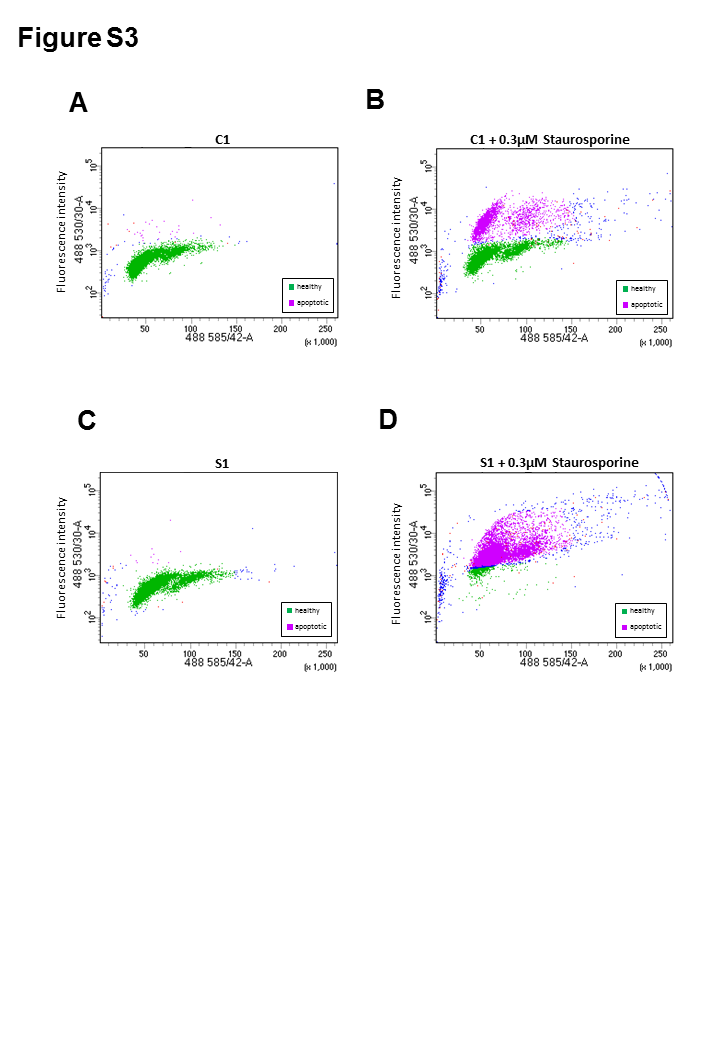

Supplement: Supplementary file 7 — High resolution image (TIF 163 kb) [file 10545_2016_9977_MOESM4_ESM.tif]
